# Mandibular trabecular fractal dimension in individuals with type 2 diabetes and hypertension: a retrospective cross-sectional panoramic radiography study

**DOI:** 10.1186/s12903-026-09785-3

**Published:** 2026-09-02

**Authors:** Burak İncebeyaz

**Affiliations:** Department of Oral and Maxillofacial Radiology, Faculty of Dentistry, Ankara Medipol University, Ankara, Turkey

**Keywords:** Bone microarchitecture, Radiomorphometry, Jawbones, bone quality, Type 2 diabetes mellitus, Panoramic radiography

## Abstract

**Background:**

This study evaluated mandibular trabecular bone microarchitecture in individuals with Type 2 diabetes and hypertension using fractal dimension analysis on panoramic radiographs, comparing findings with healthy controls.

**Methods:**

This retrospective study included 156 individuals (Control: 43, Hypertension: 63, Diabetes: 50). Fractal dimension was measured on mandibular molar regions using the box-counting algorithm. Age-related confounding effects were adjusted for using Analysis of Covariance. Intra-observer reliability was assessed via Intra-Class Correlation Coefficient.

**Results:**

Gender distribution was similar across groups, but controls were younger. After statistical age adjustment, systemic diseases impacted fractal dimension values. Age-adjusted fractal dimension averages for the diabetes (1.409) and hypertension (1.407) groups were higher than the control group (1.386). Intra-observer reliability was excellent.

**Conclusions:**

Type 2 diabetes was significantly associated with higher microarchitectural complexity in mandibular trabecular bone, whereas the increase in the hypertension group did not reach statistical significance. Fractal analysis on routine panoramic radiographs may serve as an adjunctive non-invasive clinical tool to assess these systemic effects, though these preliminary findings require validation with established reference methods.

## Background

Although bone quality has been defined solely on bone mineral density (BMD) for many years, it is now accepted that factors such as bone microarchitecture, cycle rate, and organic matrix integrity are at least as critical as density in determining fracture risk [1, 2].

The skeletal system interacts dynamically with many metabolic and vascular processes in the body. Therefore, common systemic diseases such as Diabetes Mellitus (DM) and Hypertension (HT) can disrupt the homeostasis of bone tissue, leading to a clinical picture defined as “skeletal fragility” [3, 4].

The devastating effects of diabetes on bone metabolism are based on a complex pathophysiology. In Type 1 diabetes, insulin deficiency directly suppresses osteoblast activity, while in Type 2 diabetes, the picture follows a more paradoxical course. Although BMD values in type 2 DM patients are normal or even higher than in control groups, it is known that bone quality deteriorates and the risk of fracture increases in these patients [5, 6]. One of the main mechanisms of this situation is that Advanced Glycation End Products (AGEs) formed as a result of chronic hyperglycemia accumulate in the bone collagen structure and reduce the elasticity of the matrix [7, 8]. Furthermore, it has been reported that an increase in sclerostin levels in diabetic individuals slows bone formation by inhibiting the Wnt signaling pathway, and microvascular damage impairs bone perfusion [9, 10].

Hypertension, on the other hand, affects bone health, especially through calcium homeostasis. It has been determined that the increase in blood pressure leads to hypercalciuria by reducing calcium reabsorption in the renal tubules, and the secondary negative calcium balance triggers the release of parathyroid hormone (PTH) [11, 12]. Chronically elevated PTH levels increase osteoclastic bone resorption, leading to weakening of the trabecular structure [13]. In the literature, it has been emphasized that high blood pressure is directly related to bone mass loss, especially in postmenopausal women [12].

The jawbones are important anatomical regions where systemic bone changes are reflected. In particular, the molar region of the mandible is one of the areas where bone adaptation and microarchitectural changes can be observed most clearly due to exposure to chewing forces [14]. However, traditional radiological methods and visual analysis of panoramic radiographs are sometimes insufficient to detect micro-level deterioration in the trabecular structure. At this point, Fractal Analysis (FA) offers the opportunity to objectively evaluate bone quality by expressing complex geometric patterns with a numerical value called “Fractal Dimension” (FD) [14, 15]. This analysis using the box-counting algorithm mathematically models the complexity and hollow structure in bone trabeculation [16, 17].

Although there are studies in the literature examining the effects of diabetes [18, 19] and hypertension [20, 21] on the jawbone separately, data directly comparing the effect of these two common systemic diseases on bone microarchitecture in the mandibular molar region with fractal analysis are limited. The main aim of this study was to determine the effects of the presence of diabetes and hypertension on mandibular trabecular bone structure by FD measurements and to reveal the radiological value of these systemic conditions in predicting bone quality.

## Methods

### Research design and ethical approval

This study was carried out with a retrospective cross-sectional design to examine the effects of systemic diseases on mandibular trabecular bone microarchitecture. This study was approved by the local institutional ethics committee (Approval date: 21.10.2025, No: 194) in full compliance with the principles of the Declaration of Helsinki. An informed consent form was obtained from all participants for routine diagnostic procedures and academic use of the data before the extraction. This study was reported in accordance with the STROBE (Strengthening the Reporting of Observational Studies in Epidemiology) guidelines.

### Determination of working groups

The sample size of the research was determined by power analysis using G*Power (Version 3.1.9.4) software. When the effect size was determined as Cohen’s f = 0.25 (medium effect for ANOVA/ANCOVA) (based on similar previous fractal analysis studies [6, 9]), the alpha error probability as 0.05, and the power (1-beta) as 0.80, it was determined that there should be a minimum of 53 participants for each group (total required sample size of 159). In the actual sample, strict exclusion criteria limited the healthy control group to 43 eligible individuals from the retrospective archive. Consequently, although the total sample size reached 156 (falling slightly short of the ideal 159) and two of the groups contained fewer than 53 participants, the disease groups were slightly oversampled (Hypertension: 63, Diabetes: 50) to maintain overall statistical power as much as possible. Participants were divided into three groups based on the following criteria. To isolate the independent effects of each condition, the study groups were strictly mutually exclusive; diabetic patients did not have a history of hypertension, and hypertensive patients did not have diabetes.

#### Diabetes Group (DM)

Patients with a disease duration of at least 5 years based on patient self-report, and whose stable glycemic control (HbA1c levels < 7.0% within the last 6 months) was confirmed via physician notes.

#### Hypertension Group (HT)

Individuals who have been receiving antihypertensive treatment (angiotensin-converting enzyme (ACE) inhibitors, calcium channel blockers, etc.) with a diagnosis of primary hypertension for at least 5 years.

#### Control group

Healthy individuals who do not have any systemic disease and do not use systemic drugs.

### Inclusion and exclusion criteria

Adult individuals (≥ 18 years of age) were included in the study. Although the control group included younger adults to establish a baseline, the disease groups predominantly consisted of middle-aged and older individuals, which reflects the natural demographic distribution of Type 2 Diabetes Mellitus and primary hypertension. Exclusion criteria include drug use that directly affects bone metabolism (bisphosphonates, chronic corticosteroids), uncontrolled endocrine disorders, previous surgical operation in the mandibular region, or the presence of intraosseous pathology, severe periodontal bone loss, or periapical lesions in the ROI region. Additionally, radiographs with motion artifacts, poor contrast, or incorrect patient positioning were excluded.

### Radiographic imaging protocol

Digital panoramic images were obtained with the Castellini X-Radius Compact digital panoramic system (Castellini, Bologna, Italy). Exposure parameters are optimized in the range of 60–85 kVp and 4–15 mA according to the patient’s physical structure through the device’s automatic morphology recognition technology (MRT). The images were taken by a single technician on patients positioned with the Frankfort horizontal plane parallel to the ground to prevent distortion.

### Fractal analysis process

The fractal analysis was performed using ImageJ software (Version 1.54, National Institutes of Health, Bethesda, MD, USA), following a customized automated macro protocol to ensure the standardization and reproducibility of the image processing steps.

#### ROI selection

Bilateral ‘Regions of Interest’ (ROIs) of 30 × 30 pixels were selected from both the right and left interdental regions between the roots of the mandibular second premolar and first molar. The fractal dimension values obtained from both sides were then averaged to yield a single mean FD value for each patient. If one side presented with artifacts or missing teeth, only the unaffected side was evaluated. To standardize the placement, the superior border of the ROI was consistently positioned 1–2 mm apical to the root apices to strictly avoid the periodontal ligament space. This relatively small and specific ROI size was deliberately chosen to ensure that the selected area consisted purely of trabecular bone, strictly avoiding the lamina dura, periodontal ligament space, and root surfaces [14], which could otherwise falsely alter the fractal dimension values. The specific 30 × 30 pixel dimension was a study-specific methodological choice to fit the available trabecular space.

#### Image processing and segmentation

**Fig. 1:**
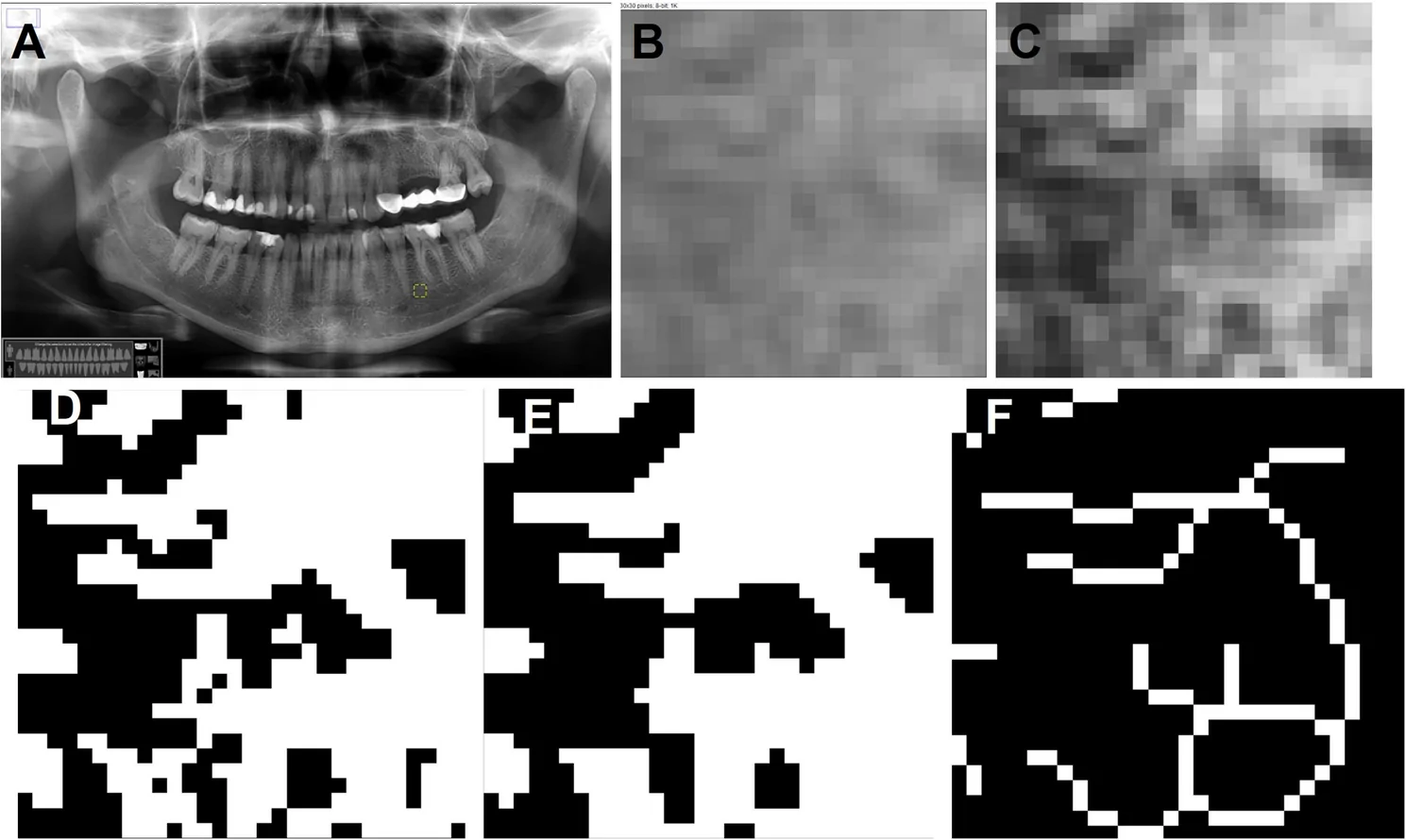
Sequential steps of the image processing protocol: (**a**) Panoramic radiograph with 30 × 30 pixel ROI, (**b**) 8-bit cropped ROI, (**c**) Contrast enhancement using CLAHE, (**d**) Binarization via Phansalkar thresholding, (**e**) Noise reduction with Despeckle, (**f**) Final skeletonized image for box-counting analysis

First, the selected ROI was cropped from the panoramic image and converted to 8-bit grayscale. Subsequently, to optimize the visibility of the trabecular meshwork, Contrast Limited Adaptive Histogram Equalization (CLAHE) was applied to the cropped ROI (blocksize = 127, histogram = 256, maximum = 3) to correct uneven illumination and enhance the local contrast of trabecular structures. Then, the image was binarized using the ‘Phansalkar’ auto local thresholding method (radius = 15), which is particularly advantageous for low-contrast biomedical images as it effectively distinguishes thin and porous trabeculae from the background better than global thresholding methods [22]. To eliminate high-frequency noise and parasitic pixels, a ‘Despeckle’ filter was applied, followed by the ‘Skeletonize’ process, which reduced the trabecular structures to a one-pixel-wide line representation (Fig. 1).

#### FD calculation

The FD value was calculated numerically using the ‘box-counting’ algorithm. To ensure compatibility with our 30 × 30 pixel ROI dimensions, the square tile sizes were limited to a range of 2 to 16 pixels (e.g., 2, 3, 4, 6, 8, 12, and 16). The FD was calculated as the absolute value of the slope of the log-log regression line, with R² values consistently > 0.95. The standard ImageJ Fractal Box Count default parameters (black background, standard grid positioning) were utilized without additional offset.

### Statistical evaluation

The data obtained were analyzed with Statistical Package for the Social Sciences (SPSS) (Version 26.0, IBM) software. The distribution of the data was controlled with the Shapiro-Wilk test. In the comparison of FD values between groups, One-Way Analysis of Variance (ANOVA) or Kruskal-Wallis H tests were used according to the normality of the distribution. To adjust for the age covariate, Analysis of Covariance (ANCOVA) was pre-specified in the analysis plan, followed by Bonferroni-adjusted post-hoc pairwise comparisons. Prior to conducting ANCOVA, the fundamental assumptions including homogeneity of regression slopes, linearity of the covariate, normality of residuals, and variance homogeneity were verified and met. For the reliability of the measurements, 20 randomly selected radiographs were reanalyzed after 15 days and the Intraclass Correlation Coefficient (ICC) (two-way mixed-effects model, absolute agreement) was calculated [23]. The significance level was accepted as *p* < 0.05.

## Results

### Analysis of demographic data

Within the scope of the study, panoramic radiographs of a total of 156 individuals were retrospectively examined. Distribution of participants by groups; Control group (*n* = 43), Hypertension group (*n* = 63) and Diabetes group (*n* = 50). There was no statistically significant difference between the groups in terms of gender distribution. In terms of mean age, a statistically significant difference was found between the groups. Post-hoc analyses showed that this difference was significantly higher than that of the control group (32.83 ± 15.23) compared to the other two study groups (Hypertension: 57.11 ± 9.13; Diabetes: 57.86 ± 8.71) was significantly younger. The specific age ranges (minimum–maximum) were 18–69 years for the control group, 41–82 years for the hypertension group, and 38–82 years for the diabetes group. Demographic data for the groups are summarized in Table 1.

**Table 1:** Distribution of groups according to demographic data

Variable	Control (*n* = 43)	Hypertension (*n* = 63)	Diabetes (*n* = 50)	Test Value	*p* Value
Age (Avg ± SD)	32.83 ± 15.2^a^	57.11 ± 9.13^b^	57.86 ± 8.71^b^	H = 58.53	< 0.001 *
Age Range (Min-Max)	18–69	41–82	38–82	-	-
Gender: Male	24 (55.8%)	29 (46.0%)	28 (56.0%)	Chi-square = 1.47	0.479
Gender: Female	19 (44.2%)	34 (54.0%)	22 (44.0%)		

*Avg* Average, *SD* Standard Deviation, *H *Kruskal-Wallis test statistic

* *p* < 0.05 was considered statistically significant

a, b: The difference between groups with different letters on the same line is significant (Kruskal-Wallis, Chi-square)

### Intra-observer reliability

In order to assess the consistency of the measurements, 20 radiographs, representing approximately 13% of the total sample, were randomly selected and re-analyzed by the same observer two weeks after the initial evaluation. The agreement between the first and second measurements was tested with the Intraclass Correlation Coefficient (ICC) and an excellent level of reliability was found (Table 2).

**Table 2:** Intra-observer reliability analysis for fractal dimension measurements

Parameters	Measurement Time	Avg ± SD	ICC (95% CI)
Fractal Dimension	1. Measurement	1.401 ± 0.034	0.942 (0.891–0.974)
Fractal Dimension	2. Measurement	1.398 ± 0.031	0.942 (0.891–0.974)

*ICC* Intraclass Correlation Coefficient, *CI* Confidence interval

*p *< 0.001 for all

### Comparison of fractal analysis data

Prior to ANCOVA, the fundamental assumptions were robustly verified: homogeneity of regression slopes was confirmed via the age × group interaction (F = 0.101, *p* = 0.904), normality of residuals was met (Shapiro-Wilk W = 0.991, *p* = 0.480), and variance homogeneity was satisfied (Levene’s test W = 0.539, *p* = 0.584).

In order to compensate for the potential confounding effect of the significant age difference between the groups on bone microarchitecture, age was included as a covariate and Analysis of Covariance (ANCOVA) was applied while analyzing the fractal dimension data. When the age variable was statistically controlled, it was determined that the presence of systemic disease (diabetes and hypertension) had a statistically significant effect on mandibular molar region FD values. When the age-adjusted (marginal) averages were examined; Pairwise comparisons revealed that the FD values of the diabetes group (1.409) were significantly higher than the control group (1.386, *p* = 0.048), whereas the difference between the hypertension group (1.407) and the control group did not reach statistical significance (*p* = 0.081). Data on observed and age-adjusted FD values are presented in Table 3.

**Table 3:** Observed and age-adjusted fractal dimension averages of the groups

Group	Observed Averages (Avg ± SD)	Adjusted Means (Marginal Means)	ANCOVA Results	Pairwise Comparisons (Bonferroni)
Control	1.393 ± 0.038	1.386 (95% CI: 1.372–1.400)	F(2, 152) = 3.14	vs. HT: *p* = 0.081
Hypertension	1.404 ± 0.036	1.407 (95% CI: 1.397–1.416)	*p* = 0.046 *	vs. DM: *p* = 1.000
Diabetes	1.406 ± 0.030	1.409 (95% CI: 1.399–1.420)	ηₚ² = 0.040	vs. Control: *p* = 0.048 *

ANCOVA: Age used as a covariate

* *p* < 0.05 was considered statistically significant

ηₚ² = 0.040: Partial eta-squared (effect size)

## Discussion

This study evaluated the effects of Type 2 diabetes (DM) and hypertension (HT), which are common in the population, on mandibular trabecular bone microarchitecture by “box-counting” fractal analysis (FD) method on panoramic radiographs. The main finding of this study was that when the age factor was statistically controlled for (ANCOVA), diabetes significantly increased bone complexity (FD value) in the mandibular molar region compared to the control group, while the observed increase in hypertension was not statistically significant. These results support the hypothesis that these systemic conditions impair bone quality independently of bone mineral density (BMD) and result in a more complex, irregular, and fragmented network in the trabecular structure [24].

The high FD value detected in the diabetic group is directly related to the condition defined as the “diabetic bone paradox” in the literature [2, 25]. Although BMD values are normal or paradoxically high in patients with type 2 diabetes, it is known that bone quality deteriorates and the risk of fracture increases in these patients [3, 5]. The main pathophysiological mechanism behind this condition is the accumulation of Advanced Glycation End Products (AGEs) formed as a result of chronic hyperglycemia in the bone collagen matrix [7, 8]. AGEs establish pathological cross-links on type I collagen fibers, reducing the biomechanical flexibility of the matrix and causing a rougher, fragmented, and complex trabecular network to form in bone tissue at the microscopic level [7]. The high FD values in this study can be considered as a radiological reflection of this structural complexity.

There are conflicting findings in the literature regarding the effects of diabetes on FD. Kurşun-Çakmak and Bayrak [6] directly evaluated panoramic FD in patients with T1DM and T2DM. Importantly, in contrast to the present study, they found no significant differences in FD between patients with T2DM and controls. They also reported an association between mandibular cortical width (MCW) and FD. These findings suggest that the higher FD observed in the present study should be interpreted cautiously, providing a more balanced interpretation of structural changes. On the other hand, Tassoker et al. [9] reported higher FD values in patients with Type 1 diabetes, although extending these findings to the complex pathophysiology of Type 2 diabetes requires separate investigation. The high FD values obtained in present study indicate that diabetes creates a structural complexity in the mandibular trabeculation and this condition can be objectively detected by fractal analysis. In addition, the fact that increased sclerostin levels in diabetic individuals slows down bone formation by inhibiting the Wnt signaling pathway supports the biochemical basis of this structural deterioration [10].

The high FD value observed in the hypertension group confirms the systemic effects of high blood pressure on bone homeostasis [11]. Hypertension reduces calcium reabsorption in the renal tubules, leading to hypercalciuria and thus a secondary negative calcium balance [12, 26]. The chronic parathyroid hormone (PTH) elevation triggered by this condition increases osteoclastic bone resorption, leading to weakening of the trabecular structure and increased porosity [12]. The micro-cavities in the trabecular bone network and the thinning and fragmentation of the trabeculae resulting from increased resorptive activity increase the geometric complexity (and therefore the FD value) of the structure.

Asantogrol and Koruyucu [13] reported that hypertension-related changes affect FD values. On the other hand, Serindere et al. [17] stated that FD values may be affected differently in some systemic diseases. These discrepancies are likely to be based on the different effects of the specific drug groups used by patients (e.g., the bone-protective effect of calcium channel blockers) and the duration of disease on bone microarchitecture [27]. Although differences in antihypertensive medications (e.g., calcium channel blockers versus ACE inhibitors) might theoretically influence bone remodeling [27], specific sub-group analyses based on medication types were not performed in this study due to sample size constraints. Therefore, the independent effects of distinct drug classes on FD values remain to be fully elucidated in future studies. Fabris et al. [20] proved the morphological effect of this condition on the jaw bones by detecting histologically increased resorption areas in the alveolar bone of hypertensive patients.

Furthermore, it is crucial to recognize that systemic bone alterations do not necessarily manifest in a single direction or consistently as increased FD. For instance, Göller Bulut et al. [28] reported that while mean FD did not differ significantly in patients using aromatase inhibitors, significantly lower FD values were observed in specific ROIs (FD2 and FD4), indicating that FD findings may be ROI-dependent. Similarly, Ustaoğlu et al. [29] found significantly lower FD values in several regions and for the mean FD in patients using antiepileptic drugs compared with controls. These findings highlight that the interpretation of ‘higher FD = structural deterioration’ in the present manuscript may not be universally applicable, as bone microarchitecture responses can vary depending on the specific systemic factor and the anatomical region analyzed.

The mandibular molar region was chosen as the site of interest (ROI) in this study because it is one of the areas most intensely exposed to masticatory forces [14]. Unlike traditional radiological indices, fractal analysis is a non-invasive method that can numerically express the geometric complexity in the internal structure of bone [14]. The processing of the age factor as a covariant (ANCOVA) in this study partially adjusted for the measured effect of age on bone microarchitecture. The high within-observer agreement (ICC: 0.942) reinforces the clinical reliability of the method.

While these findings demonstrate a more complex and fragmented trabecular pattern in patients with diabetes and hypertension, they should be interpreted strictly as radiographic associations rather than direct evidence of clinical ‘skeletal fragility’. Because this study did not clinically evaluate fracture risk, primary implant stability, osseointegration success, or specific surgical outcomes, drawing definitive clinical guidelines from these radiographic measurements is premature. Furthermore, recent literature highlights that mandibular trabecular architecture and panoramic radiomorphometric measurements are highly sensitive not only to systemic conditions but also to local dental conditions and longitudinal changes. For instance, local anatomical factors (such as periodontal status and tooth loss) and the temporal progression of systemic diseases can significantly influence fractal measurements [30, 31]. Therefore, while the microstructural deterioration detected by box-counting analysis might theoretically influence biomechanical bone properties, further prospective clinical studies are required to determine if these radiographic changes translate to a higher risk for micro-fractures or compromised implant healing.

This study has some limitations. The most important limitation was that the mean age of the control group (32.83 ± 15.2) was significantly lower than that of the hypertension (57.11 ± 9.13) and diabetes (57.86 ± 8.71) groups. It is known that bone structure enters a natural process of change and loss with age. In order to eliminate the potential confounding effect of this situation on the results, the age factor was treated as a covariant in the statistical analysis process and the ANCOVA test was applied. Thanks to this method, the measured effect of age on the mandibular trabecular bone could be partially adjusted. To further address the limited age overlap, a sensitivity analysis restricted to a shared age range (41–69 years, *n* = 115) was performed. Although the age-adjusted FD means exhibited a similar trend (Control: 1.385, Hypertension: 1.406, Diabetes: 1.408), the overall ANCOVA lost statistical significance (*p* = 0.136), primarily due to the severe reduction in the control group sample size (*n* = 12). This highlights the fundamental limitations of the retrospective sample distribution. In addition, due to the retrospective design of the study, several important clinical confounders could not be adequately assessed. Detailed data regarding specific antihypertensive medication classes, the exact continuous numerical values of recent HbA1c and exact disease duration (despite categorical confirmation of eligibility in their medical records), body mass index (BMI), smoking habits, menopausal status, and local factors such as periodontal condition and extent of tooth loss were incomplete or unavailable. Consequently, we were unable to perform adjusted analyses for these potentially confounding variables. Furthermore, a major limitation is the absence of validation with an established reference method, such as Dual-energy X-ray Absorptiometry (DXA) or micro-computed tomography (micro-CT). Because statistical corrections like ANCOVA cannot fully compensate for fundamental baseline imbalances in retrospective designs, future prospective studies incorporating these detailed clinical characteristics and reference imaging techniques are essential to isolate the independent effects of these systemic diseases on bone microarchitecture. Moreover, moving beyond traditional box-counting algorithms, future investigations could utilize advanced panoramic radiomics and sophisticated texture analysis to track mandibular microarchitecture. Such high-dimensional data extraction could provide a more robust and comprehensive evaluation of bone quality, potentially mitigating some of the methodological limitations associated with conventional fractal dimension measurements.

Finally, it should be acknowledged that absolute FD values are highly sensitive to the specific image-processing parameters applied, such as ROI size, spatial resolution, and the choice of local thresholding algorithms (e.g., Phansalkar). Therefore, comparisons of absolute FD values across different studies should be interpreted with caution, as variations in image-processing choices can inherently affect the resulting fractal dimensions.

## Conclusions

In conclusion, this study revealed that the fractal analysis method applied on panoramic radiographs is a sensitive tool in detecting the effects of common systemic diseases such as Type 2 diabetes and hypertension on mandibular bone microarchitecture. The findings indicate that diabetes significantly increases trabecular bone complexity (FD value) in the mandibular molar region compared to the control group, whereas the increase in the hypertension group was not statistically significant. The high FD values observed in diabetic individuals demonstrate a radiographic association between this systemic condition and increased trabecular complexity. While these elevated FD values may theoretically reflect structural deterioration (such as the “diabetic bone paradox”) and increased resorptive activity, they should not be interpreted as direct evidence of clinical skeletal fragility. Therefore, “box-counting” fractal analysis performed on routine panoramic radiographs serves as a non-invasive, adjunctive method for evaluating bone microarchitecture changes. However, given the retrospective nature of the study, the unmeasured clinical confounders, and the lack of validation against established reference methods, these findings should be considered preliminary. Future studies evaluating actual clinical outcomes, such as implant stability and fracture rates, along with reference standards like DXA, are necessary before utilizing this method as a definitive risk assessment tool in oral surgery protocols. 

## Data Availability

“The dataset(s) supporting the conclusions of this article is (are) included within the article (and its additional file(s)). Additional raw data and analysis files generated during the current study are available from the corresponding author upon reasonable academic request.”
